# Deterministic Fabrication of Fluorescent Nanostructures Featuring Distinct Optical Transitions

**DOI:** 10.3390/nano15030219

**Published:** 2025-01-29

**Authors:** Marijn Rikers, Ayesheh Bashiri, Ángela Barreda, Michael Steinert, Duk-Yong Choi, Thomas Pertsch, Isabelle Staude

**Affiliations:** 1 Institute of Solid State Physics, Friedrich Schiller University Jena, Max-Wien-Platz 1, 07743 Jena, Germany; marijn.rikers@uni-jena.de (M.R.); ayesheh.bashiri@uni-jena.de (A.B.); angela.barreda@uni-jena.de (Á.B.); 2 Institute of Applied Physics, Abbe Center of Photonics, Friedrich Schiller University Jena, Albert-Einstein-Str. 15, 07745 Jena, Germany; michael.steinert@uni-jena.de (M.S.); thomas.pertsch@uni-jena.de (T.P.); 3 ARC Center for Transformative Meta Optics, Department of Quantum Science and Technology, Research School of Physics, Australian National University, 60 Mills Rd., Canberra, ACT 2601, Australia; duk.choi@anu.edu.au; 4 Department of Electronic Engineering, University Carlos III of Madrid, Avda. de la Universidad 30, 28911 Leganés, Spain; 5 Fraunhofer-Institute for Applied Optics and Precision Engineering IOF, Albert-Einstein-Str. 7, 07745 Jena, Germany; 6 Max Planck School of Photonics, Hans-Knöll-Str. 1, 07745 Jena, Germany

**Keywords:** nano-fabrication, localized emitters, Eu^3+^, magnetic dipole transitions, electron beam lithography

## Abstract

The precise and deterministic integration of fluorescent emitters with photonic nanostructures is an important challenge in nanophotonics and key to the realization of hybrid photonic systems, supporting effects such as emission enhancement, directional emission, and strong coupling. Such integration typically requires the definition or immobilization of the emitters at defined positions with nanoscale precision. While various methods were already developed for creating localized emitters, in this work we present a new method for the deterministic fabrication of fluorescent nanostructures featuring well-defined optical transitions; it works with a minimal amount of steps and is scalable. Specifically, electron-beam lithography is used to directly pattern a mixture of the negative-tone electron-beam resist with the europium complex Eu(TTA)_3_, which exhibits both electric and magnetic dipolar transitions. Crucially, the lithography process enables precise control over the shape and position of the resulting fluorescent structures with a feature size of approx. 100 n m . We demonstrate that the Eu(TTA)_3_ remains fluorescent after exposure, confirming that the electron beam does not alter the structure the optical transitions. This work supports the experimental study of local density of optical states in nanophotonics. It also expands the knowledge base of fluorescent polymer materials, which can have applications in polymer-based photonic devices. Altogether, the presented fabrication method opens the door for the realization of hybrid nanophotonic systems incorporating fluorescent emitters for light-emitting dielectric metasurfaces.

## 1. Introduction

Quantum emitters can emit light via the process of spontaneous emission [1]. This effect is observed in atoms and molecules, quantum dots, quantum wells, and defect centers in crystals. Importantly, the emission rate of a given transition is not fixed but depends on the electromagnetic environment of the source. While this effect was first described by E.M. Purcell in 1946 in the context of nuclear magnetic resonance [2], it is now at the heart of an important research stream in nanophononics aiming to control the emission properties on nanoscale light sources using engineered photonic nanostructures.

Up to now, most research on manipulating spontaneous emission with nanostructures has focused on electric dipole (ED) transitions. Magnetic dipole (MD) transitions are usually several orders of magnitude weaker and thus often neglected [3]. However, there is a growing interest in the study of magnetic light–matter interactions [4], which is facilitated by the deployment of special quantum emitters, such as rare-earth ions [5,6,7] or semiconductor quantum dots [8]. These emitters can feature prominent MD transitions with comparable or even greater strength than their ED transition [9]. Various photonic structures have been investigated for their potential to enhance the MD transition, including metallic mirrors, metal films, hyperbolic metamaterials, and a range of plasmonic and all-dielectric nanostructures [8,10,11,12,13,14,15,16,17,18,19,20,21,22,23]. Low-loss high-refractive-index dielectric nanostructures are particularly interesting for coupling with magnetic emitters. Despite the non-magnetic response of the constituent dielectric materials (μ=1) , they exhibit magnetic Mie-type resonances [24], even for basic structure geometries such as spheres or cubes [25,26,27,28]. Moreover, they can be engineered to support high-quality-factor Fano resonances or quasi-bound states in the continuum [29]. Such resonances are often accompanied by significant magnetic field enhancements, which can reach several orders of magnitude for specifically tailored photonic structure geometries [30,31]. Therefore, high-refractive-index dielectric nanostructures show great promise in tailoring the emission of magnetic dipole transitions through the magnetic Purcell effect.

Experimental demonstrations so far include the modification of the branching ratio of emission via the electric or magnetic transition channel using, e.g., a Mie-resonant dielectric metasurface [18] and the enhancement of magnetic light emission with all-dielectric optical antennas [20]. However, in almost all realized coupled systems, there has been no control over the lateral placement of the emitters. Typically, the emitters are dissolved in a polymer and spin-coated onto the prefabricated nanostructures [14,19]. This results in a layer of active material covering the nanostructures. A notable exception was presented by Sugimoto et al. [32], where the branching ratio between the magnetic and electric dipole transitions was enhanced by a factor of up to 7 using a colloidal silicon nanosphere antenna decorated with Eu^3+^ complexes. However, the employed wet chemical process is very limited in terms of the accessible geometries and does not allow for a selective decoration of only certain parts of the sphere’s surface. Sanz-Paz et al. obtained positional control by defining the nanoantennas at the tip of a scanning probe microscope [20]. While this method is powerful for basic studies, it is not suitable for fabricating integrated photonic quantum systems.

The lack of control over the placement of the emitters in integrated nanostructure geometries largely limits the performance and functional scope of the coupled systems. Precisely placing the emitters in the photonic structure architecture is crucial for effectively controlling the coupling between the two entities [33]. Ideally, for maximum interaction, the emitter should be positioned in the area where the magnetic local density of optical states (LDOS) takes the largest values [25]. Alternatively, precise positioning of the emitters can enable control over the directional emission properties [34]. The target areas for emitter placement typically have subwavelength dimensions, posing a challenge for fabricating the hybrid structures with the required precision. For ED emitters, a range of methods for their precise placement and integration with photonic nanostructures has been demonstrated [35], including selective surface functionalization in combination with covalent binding of the emitters [36,37], dip-pen lithography [38], local exposure of emitter-doped resists [39], nanomanipulation with an atomic force microscopy (AFM) tip [40], and fabrication of nanostructures at predetermined emitter positions [41]. However, the flexible and deterministic fabrication of nanoscale photonic architectures with the incorporation of precisely placed fluorescent material remains challenging. For example, Ref. [39] uses a positive resist, which is not suitable for the small filling factor exposures needed to define nanoscale emitter structures. The AFM method proposed by [40] allows for the study of single nanoantennas, but does not scale to metasurfaces. Dip-pen lithography suffers from limited resolution and determinism of the structures, the fabricated spots are > 500 nm wide and vary in shape [38]. Selective surface functionalization can yield deterministic structures with accuracies below 100 nm [36]. However, this approach requires multiple chemical processes and is highly substrate dependent. C. A. Barrios (2012) [42] and H. M. Lee (2008) [43] demonstrated the fabrication of nanoscopic fluorescent structures using image reversal in the electron-beam resist polymethyl methacrylate (PMMA). By using electron-beam exposures with a high dose, $>1mC\;cm^{-2}$ , the normally positive PMMA resist becomes both negative and fluorescent. The resulting fluorescence spectra are very broad (>100 nm linewidth). Most importantly, the precise placement of emitters featuring magnetic dipolar transitions has not been achieved so far. In contrast, our work realizes a negative-tone resist with embedded Eu(TTA)_3_ emitters featuring well-defined electric and magnetic dipolar transitions and corresponding distinct peaks in their fluorescence spectra.

**Figure 1:**
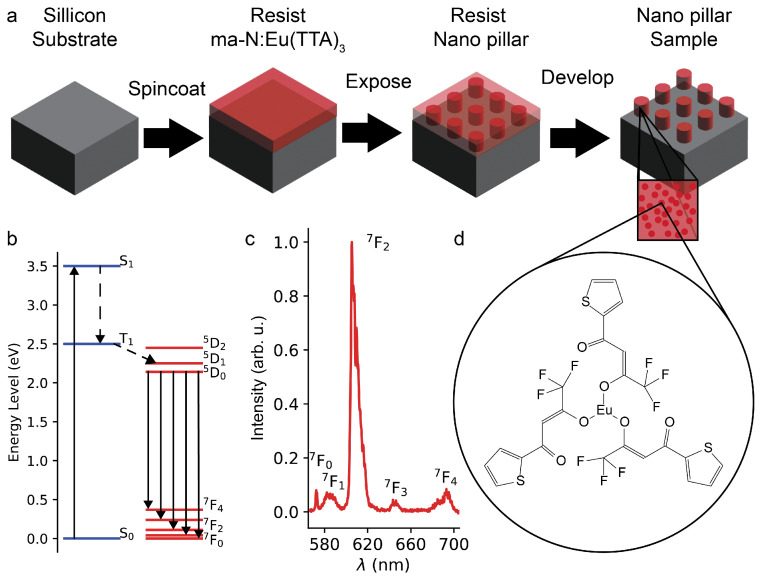
(**a**) Process of creating the fluorescent nanostructures, from left to right. First, the resist is spin-coated onto the substrate. Next, it is exposed with electron-beam lithography. Third, it is developed to obtain the fluorescent nanostructures. (**b**) Energy diagram of Eu(TTA)_3_, where the ligand absorbs UV light (up arrow). Then, by non-radiative (dashed arrows) energy transfer, the ^5^D_0_ state is populated, and by radiative decay (indicated with solid arrows) the photons with different energies are emitted. (**c**) Fluorescence spectra of unexposed ma−N:Eu(TTA)_3_ thin film (100 n m ) with a Eu(TTA)_3_ concentration of 1% . (**d**) Chemical structure of Eu(TTA)_3_.

Specifically, we demonstrate the deterministic fabrication of fluorescent nano- and microstructures by directly exposing a Eu^3+^-doped resist with electron-beam lithography, as shown in Figure 1a. The fabrication scheme thus inherits the full structural flexibility and placement accuracy from the employed electron-beam lithography (EBL) process, including the capability for precise integration into nanostructures via precision-aligned two- or multistep EBL procedures [44]. For a more comprehensive review of EBL we refer the reader to review papers by A. A. Tseng [45] or Y. Chen [46]. Eu^3+^ is particularly suitable for studying MD transitions as the Eu^3+^(^5^D_0_) ⟶ Eu^3+^(^7^F_1_) transition occurs in the visible wavelength ( λ ) at λ=590 nm . Eu^3+^ also features several ED transitions, with Eu^3+^(^5^D_0_) ⟶ Eu^3+^(^7^F_2_) at λ=610 nm being the dominant one [47].

To obtain the Eu^3+^-doped resist, europium complexes Eu(TTA)_3_ are embedded into a negative-tone EBL resist (ma−N 2400), which works as a host matrix. As shown in Figure 1d, Eu(TTA)_3_ is selected because its organic chains allow the complex to dissolve effectively in common solvents [48]. The complexes are excited in the UV spectral range ( λ<375 nm ). Through non-radiative processes, the absorbed energy is transferred to the ionic core of the complex, where the emission occurs for Eu^3+^(^5^D_0_) ⟶ Eu^3+^(^7^F_*i*_) i∈{0,1,2,3,4} ; see Figure 1b. The emissions from the complexes are from an “ionic” state, resulting in sharp transitions with a full-width at half maximum (FWHM) of 10 nm in the visible region; see Figure 1c.

This approach is directly scalable up to wafer-level processing within the boundaries of electron-beam lithography as its base technology, making it highly suitable for scientific applications and prototyping. However, for industrial applications that require series or mass production of nanostructured wafers, additional research and approaches, e.g., involving UV lithography, would be required. Altogether, our method opens the pathway for the experimental realization of designed photonic nanostructures incorporating localized nanoscale emitters. In particular, this top-down approach offers the opportunity to create both single fluorescent objects and deterministically placed arrangements of many nano-objects. Thus, our work will enable the realization of previously inaccessible designs of integrated hybrid quantum systems, thereby empowering the deeper study of light–matter interactions that rely on the precise positioning of the emitters in the nanostructure.

## 2. Materials and Methods

The transitions of Eu^3+^ are very well defined, making them a good candidate for probing the LDOS of nanostructures. Unlike fluorescent films that cover a complete array, randomly dispersed nanoparticles without control, or complicated chemical procedures, here we demonstrate the deterministic fabrication of fluorescent emitters on the scale of metasurfaces, with the resolution of meta-atoms in the visible.

To prepare the photoluminescent resist, Eu(TTA)_3_ (Gelest Inc., Morrisville, PA, USA: Europium(III)Thenoyltrifluoroacetonate 95% ) was mixed with the negative-tone electron-beam resist ma−N 2401 (Micro Resist Technology, Berlin, DE, Germany: ma−N 2401), with a mass percentage of 0.1, by stirring it for 10 min. The solution was stored in the dark for 24 h and subsequently filtered with a 0.2 μm filter. Next, the ma−N: Eu(TTA)_3_ mixture was spin-coated onto a 15 mm×15 mm Si substrate (500 rpm for 5 s followed by 3000 rpm for 60 s ). Then, it was baked on a hotplate at 90 °C for 60 s . Electron-beam exposure was performed at 30 keV with a current of 20 pA using the commercial EBL system (RAITH GmbH, Dortmund, De, Germany: eLINE). The exposure dose was controlled by the dwell time. Following the exposure, the substrate was developed in ma−D 331 developer for 30 s , then washed with distilled water, and dried with N_2_ gas.

It is well known that the Eu^3+^(^5^D_0_) ⟶Eu^3+^(^7^F_2_) transition (λ=610 nm) is very sensitive to the local environment [49]. It is therefore important to check if the fluorescence of the emitters is dependent on the electron-beam dose. Furthermore, as the electron-beam dose determines the state of the electron-beam resist, it is also important to investigate if there is parasitic emission from ma−N, like PMMA in [42], for the different considered electron-beam doses. Thus, as a first step, we investigated the fluorescence as a function of the electron-beam exposure dose. To this end, we fabricated arrays of microstructures exhibiting a bowtie shape with a size of 3 μm×3 μm and a gap of 100 nm , from the doped resist and varied the exposure dose between 100 μ C c $m^{-2}$ and 540 μ C c $m^{-2}$ in steps of 20 μ C c $m^{-2}$ . Next, the sensitivity, contrast, and resolution limit of the resist were investigated.

## 3. Results

### 3.1. Fluorescence of Exposed Doped Resist

**Figure 2:**
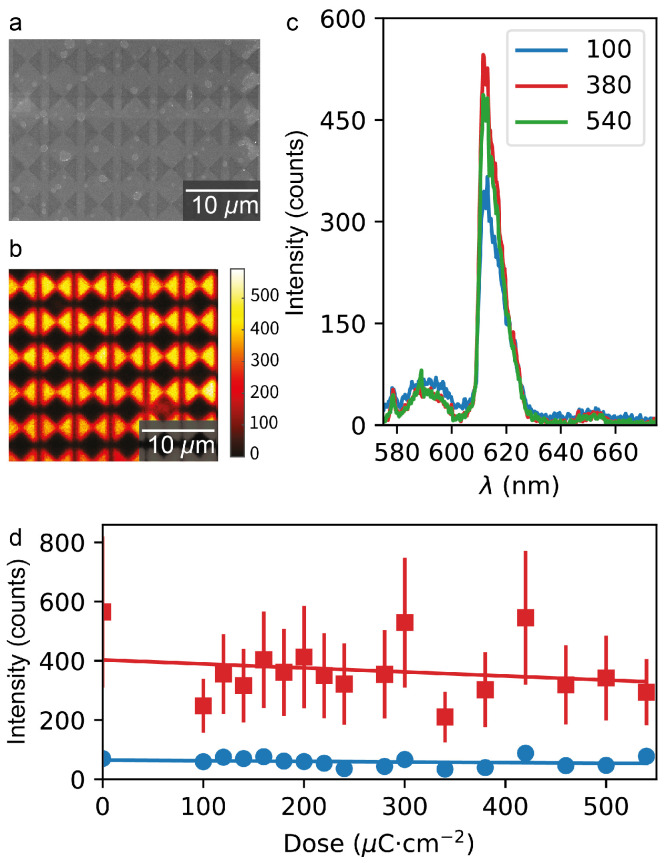
(**a**) SEM of microscopic bowtie array fabricated from the ma−N:Eu(TTA)_3_ resist mixture at an exposure dose of 380 μ C c $m^{-2}$ . (**b**) Fluorescence map of the same resist structures as shown in (**a**). (**c**) Fluorescence spectra taken from microscopic bowties similar to these shown in (**a**) for different exposure doses used for fabrication. Red, green, and blue lines correspond to doses of 100, 380, and 540 μ C c $m^{-2}$ , respectively. (**d**) Intensities of the λ=590 nm transition, blue circles, and the λ=610 nm transition, red squares, for structures fabricated at different exposure doses. The solid lines are linear fits to the data.

Figure 2a shows a scanning electron micrograph (SEM) of a fabricated bowtie array exposed at a dose of 380 μ C c $m^{-2}$ . This dose resulted in the best reproduction of the structural design parameters and a gap of 100 nm. Dark areas in the image represent the resist structures. Note that there is some residual resist remaining in between the actual structures, which could likely be avoided by further optimization of the development process or additional process steps such as an oxygen plasma etch. To analyze the general fluorescence properties of the exposed structures, a commercially available confocal laser scanning microscope (PicoQuant, Berlin, Germany: MicroTime 200) was used. The samples were excited within the excitation band of Eu(TTA)_3_ with a 375 n m laser (pulse duration 60 p s , repetition rate 20 M Hz , output power of 25 μ W ). For focusing on the sample, a 0.95 NA 100× Olympus objective was used, which also served to collect the emitted light in reflection geometry. The excitation conditions resulted in an expected laser spot diameter on the sample of 250 n m . A piezo stage served to control the position of the objective. The fluorescent signal was then counted with a single-photon avalanche diode. A filter with a pass band between λ=550 nm and λ=750 nm and a longpass filter with a cut-on edge at λ=550 nm were used to remove the excitation light from the signal reaching the detector.

Figure 2b shows the measured fluorescence map of the same structure as depicted in Figure 2a. As a first important observation, the Eu(TTA)_3_ remains fluorescent after exposure to the electron beam, and the shapes of the microstructures are well reproduced in the measurement.

Next, to investigate the fluorescence spectra of the sample, the microscope output was coupled to a spectrometer (Oxford Instruments, Belfast, Northern Ireland: Andor Kymera 328i) equipped with a camera (Andor Newton) to record the signal. Figure 2c shows the measured spectra of the structures fabricated, similar to the structures in Figure 2a, with exposure doses of 100 μ C c $m^{-2}$ , 380 μ C c $m^{-2}$ , and 540 μ C c $m^{-2}$ . The signals are not corrected for background noise. Notably, while the peak intensity varies for the different structures, a systematic dependence of the fluorescence signal on the exposure dose is not apparent. Furthermore, the spectral position of the fluorescence maximum is not affected by the exposure for any of the considered dose values. Importantly, and in contrast to [42], we do not detect any parasitic emission from the resist at the excitation powers used in this experiment.

Figure 2d shows the fluorescence intensity as a function of exposure dose. Each data point is obtained from spectrally resolved measurements by averaging the signal within a 10 n m window around λ=590 nm and λ=610 nm . We then fit the resulting data using a standard line fit I(D)=aD+b . For λ=590 nm this procedure yields a=0.02,b=64.6 , and a standard deviation of σ=15 ; while for λ=610 nm we obtain a=−0.13,b=403 , and σ=93 . From this we deduce that there is a negligible change in intensity for the 590 n m transition while the 610 n m transition shows a small decrease, noting that the latter also exhibits a much larger variability in its intensity. However, there is a large variation in the intensity of the signal at λ=610 nm . For example, at a dose of 300 μ C c $m^{-2}$ , the count is 400 while at a dose of 320 μ C c $m^{-2}$ the count is 200. Furthermore, there is a reduction in intensity at λ=610 nm as compared to the unexposed resist. On average, the counts are 300 compared to 400 for the unexposed resist under otherwise-identical measurement conditions. We attribute this reduction and variability to the hyper-sensitive nature of the Eu^3+^(^5^D_0_) ⟶ Eu^3+^(^7^F_2_) transition. This transition is very sensitive to the local electronic potentials as compared to other transitions [50].

### 3.2. Deterministic Fabrication of Fluorescent Nanostructures

To further demonstrate the capability of our approach to define nanoscale fluorescent structures in a deterministic way, we fabricate two more types of example structures, namely, one complex structure with multi-scale features and an array of nanoscale dots. The complex geometry can only be created with a high-contrast resist, while the dot array demonstrates the resolution limit of the resist. The dot array is designed to have a period of 800 n m . The resolution limit of the pure ma−N 2401 resist is specified to be 50 n m by the manufacture (Micro Resist Technology, Berlin, DE: ma−N 2401).

After testing various emitter concentrations, we found that a ma−N_1−x_:(Eu(TTA)_3_)_x_ $x=9\times 10^{-4}$ , where *x* indicates the mass ratio, resulted in reasonable exposure characteristics like sensitivity and contrast, while showing sufficiently strong fluorescence for optical characterization; see Appendix A for more details. The optimal exposure dose for this mixture, which resulted in the smallest dots without showing any signs of underexposure, such as detachment from the substrate, was found to be 380 μ C c $m^{-2}$ .

**Figure 3:**
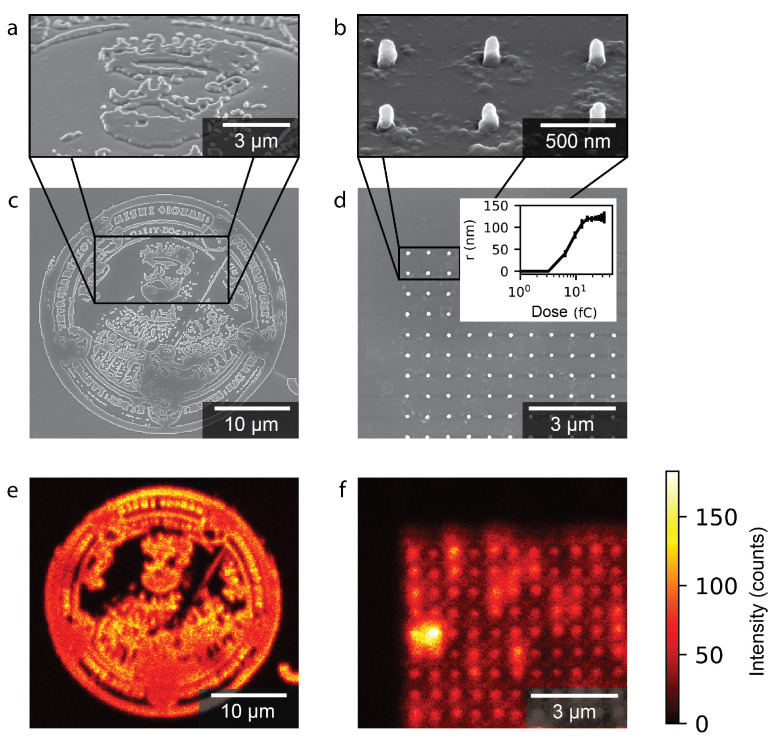
Tilted close up SEM of (**a**) the fabricated fluorescent FSU logo, and (**b**) a squared array of nanodots demonstrating ability to create complex shapes. (**c**,**d**) Top-view SEM of fabricated logo and nanodot array. (**e**,**f**) Fluorescence maps of the logo and nanodot array, respectively, with the scale bar of the intensity to the right of the nanodot array. The inset in (**d**) shows the mean radius (r) of an array of dots as a function of the exposure dose (D) .

In Figure 3a,c,e, the Friedrich Schiller University (FSU) logo is presented. The logo of 50 μ m is exposed using a 100 μ m write field with the previously mentioned optimal beam conditions. The corresponding layout is generated by making a binary from a logo image and converting it into a layout file (GDSII). The minimal feature size of the layout is 100 n m and the maximum feature is 5 μ m . These features are all well reproduced in the exposed resist. In Appendix B, a comparison between the FSU logo layout and the SEM image of the developed fluorescent resist is shown. This demonstrates that the contrast of the resist is not significantly affected by the presence of the Eu(TTA)_3_ and remains functional as a negative-tone EBL resist. The exposures were performed without utilizing a proximity correction algorithm for the exposure, which could be implemented to further improve the accuracy with which complex patterns are reproduced.

Figure 3b,d,f, summarizes the results obtained for the nannodot arrays. The nanodots demonstrate the minimal feature size possible in the resist. The tilted SEM of the resist dots in Figure 3b shows well-defined pillars with vertical walls. However, there are some residues surrounding the pillars, suggesting that the development procedure needs to be optimized. The dot array shown in Figure 3d, with corresponding fluorescence map in Figure 3f, was exposed with a dose of 5 f C . The inset in Figure 3d shows the radius *r* as a function of the dot dose *D*. This was measured by exposing an array of dots with a pitch of 400 n m to different doses, varying logarithmically from 0.32 f C to 32 f C . Next, the mean radius of the dots in the array, as observed after development, was measured using SEM images. If the dots are not present, the radius is set to zero. The dots require a dose of 5 f C to remain on the substrate for a radius of 40 n m . The radius of the dots increases to 100 n m for a dose of 15 f C . For higher doses, the radius saturates around 120 n m .

## 4. Discussion

We have demonstrated the deterministic fabrication of fluorescent nano- and microstructures featuring distinct electronic transitions. This was achieved by direct exposure of a negative-tone resist mixed with Eu(TTA)_3_ via electron-beam lithography. In particular, we created fluorescent structures, namely, dots with a radius of 40 n m . The resist also has good contrast, as demonstrated by the fabrication of the university logo that has a large feature size range from 100 n m to 3 μ m , thereby evidencing the high structural flexibility of our approach in an exemplary fashion. Using microscopic fluorescence spectroscopy, we show that the Eu(TTA)_3_ emission spectrum is not qualitatively altered by the electron beam exposure. The fluorescent resist has good exposure properties, with an optimal exposure at 380 μ C c $m^{-2}$ for area exposures and 5 f C for dot exposures.

The main challenge with our fluorescent resist arises in the development process. As seen in Figure 3, there are residues left on the sample. To reduce these residues and further improve the structure quality, further optimization of the development process is required. We already performed some initial steps in this direction. As a first step, we tested longer development times. However, in this case the adhesion of the nanostructures to the substrate weakens, causing them to detach. Thus, as a next step, we investigated the use of hexamethyldisilazane (HMDS) (Micro Resist Technology, mr-APS1) as a common adhesion promoter for Si substrates and ma−N 2400 series resists. However, this adhesion promoter did not prove effective for our fluorescent resist. While these initial steps did not yet yield the desired outcome, we are confident that further optimization of the development process is possible. For example, different developers for ma-N resists like ma-D332 or ma-D525, different adhesion promoters or substrate functionalizations, as well as additional process steps like dry etching for footage removal, can be tested.

Our method builds upon the knowledge of fluorescent electron-beam resists and improves upon previous works, now enabling a narrow linewidth of ≈10nm for distinct optical transitions (Eu^3+^(^5^D_0_) ⟶ Eu^3+^(^7^F_*i*_) i∈{0,1,2,3,4} ) of both electric and magnetic dipolar character. As such, the fluorescent nanodots demonstrated here offer new opportunities to probe both the magnetic and electric components of light, thanks to the magnetic dipolar character of the Eu^3+^(^5^D_0_) ⟶ Eu^3+^(^7^F_1_) transition. Importantly, our fabrication approach enables experimental studies up to wafer scale. Considering the high flexibility and spatial resolution of the EBL process, it also allows for the precise integration of emitters into arbitrary nanostructures via precision-aligned two- or multistep EBL procedures. As such, our work offers important new opportunities for the study of light–matter interactions at the nanoscale, enabling the experimental realization of even complex designs of integrated hybrid quantum systems that require the exact placement of emitters.

## Figures and Tables

**Figure A1:**
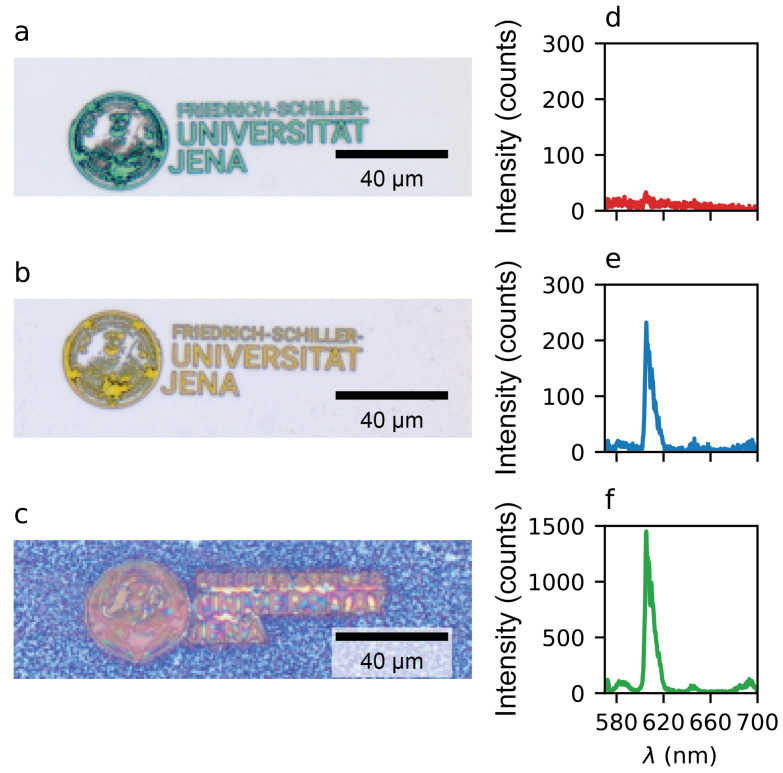
Bright-field microscope images of patterned resists with (**a**) no Eu(TTA)_3_, (**b**) an Eu(TTA)_3_ concentration of 0.1% , and (**c**) a concentration of 1% . (**d**–**f**) are fluorescence spectra taken from the logos shown in (**a**–**c**), respectively.

**Figure A2:**
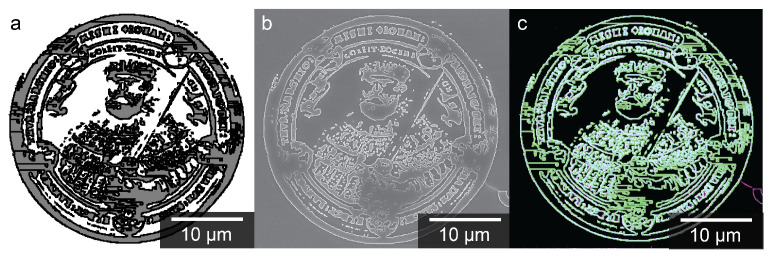
(**a**) Layout of the FSU Jena logo used as a test for the exposure of complex geometry with EBL. (**b**) SEM image of the resulting fluorescent resist mask after exposure and development. (**c**) The overlay of the SEM image shown in (**b**) where white is turned to magenta and the layout shown in (**a**) in green. There is almost no magenta remaining, meaning we have a qualitative match between layout and SEM image.

## Data Availability

Data are contained within the article.

## Abbreviations

The following abbreviations are used in this manuscript:

Eu(TTA)_3_	Europium(III) thenoyltrifluoroacetonate
ED	Electric dipole
MD	Magnetic dipole
LDOS	Local density of optical states
EBL	Electron-beam lithography
SEM	Scanning Electron Microscope
λ	wavelength

## Appendix A. Concentration in Resist

Adding Eu(TTA)_3_ to the ma−N resist significantly alters the exposure properties depending on the concentrations of the resist. In Figure A1, the effect of different Eu(TTA)_3_ concentrations is demonstrated. As a reference, a pure ma−N resist is shown in Figure A1a, with the corresponding fluorescence spectrum in Figure A1d. The optimal ma−N:Eu(TTA)_3_ mixture, with a concentration of 0.1% , is represented in Figure A1b, with the spectra in Figure A1e. In Figure A1c, with the fluorescence spectrum in Figure A1f, an Eu(TTA)_3_ concentration of 1% are provided. The resist does not develop properly at this concentration and a thin film (10 n m ) of fluorescent material is left on the sample. The spectra shown in the figures were collected under the same measurement conditions as described in the main text. With the optimal concentration of Eu(TTA)_3_, the sample develops properly and all the transition lines of the compound are detectable.

## Appendix B. Layout Compared to SEM

Here, we compare the layout of a complex geometry, in our case a binary of the FSU Jena logo, and the developed fluorescent resist. Figure A2a presents the layout, Figure A2b the developed resist after exposure, and Figure A2c the overlay of the two. To generate the overlay, the outline of the layout is rendered in green, and in the SEM image white is turned to magenta; as a result, when the two images are overlaid the result is light blue. In Figure A2c, almost no magenta remains, meaning we have a qualitative match between the layout and exposed structure. In the figure, there are some horizontal green lines which are artifacts of the generated layout which connect the paths. This does not have an effect on the exposure.
